# Heterologous Expression of the Hot Pepper ABA 8’-Hydroxylase in *Escherichia coli* for Phaseic Acid Production

**DOI:** 10.4014/jmb.2301.01014

**Published:** 2023-02-03

**Authors:** Hyun Min Kim, Young Hee Joung

**Affiliations:** School of Biological Sciences and Technology, Chonnam National University, Gwangju 61186, Republic of Korea

**Keywords:** ABA hydroxylation, cytochrome P450, CaCYP707A, Phaseic acid, heterologous expression

## Abstract

The CYP707A family genes encoding ABA 8’-hydroxylase catabolize abscisic acid (ABA), a plant stress hormone that plays an important role in stress condition, such as drought, heat, cold and salinity. Phaseic acid (PA) is a catabolic product of ABA. Recent studies have shown that PA is important for the physiological functions in plants. It is also a neuroprotective molecule that protects against ischemic brain injury in mice. To obtain enzymes for the PA production, four *CaCYP707A* genes (*CaCYP707A1*, *CaCYP707A2*, *CaCYP707A3* and *CaCYP707A4*) were isolated from hot pepper. They were heterologously expressed in *Escherichia coli*. Among them, CaCYP707A2 showed significantly higher expression levels in both the membrane fraction and the soluble fraction. Preferred redox partners were investigated to improve the efficiency of CaCYP707A2's catalytic reaction, and NADPH-cytochrome P450 reductase (CPR) from hot pepper (CaCPR) was preferred over other redox partners (*i.e.*, rat CPR and ferredoxin reductase/ferredoxin). The production of 8’-hydroxy ABA and PA by ABA hydroxylation activity was confirmed in CaCYP707A2 from both membrane and soluble fractions. Therefore, CaCYP707A2 is the first identified plant CYP protein that is expressed a soluble form in cytosolic fraction having stable activity. Taken together, we propose a new CYP707A protein with industrial applications for PA production without additional modifications in *E. coli* heterologous expression.

## Introduction

Cytochrome P450 (CYP) is a monooxygenase present in almost all living organisms, including animals, plants, and microorganisms, and is involved in various types of oxidation reactions including hydroxylation, epoxidation, dealkylation, and decarboxylation [1, 2]. CYP acts as a biocatalytic enzyme with various functions, and has the ability to selectively catalyze oxidation at the inactive C-H position of complex molecules, which are difficult to produce through chemical catalytic reactions, therefore, it can be important for metabolic engineering to use CYP [3, 4]. In particular, plant CYPs are the third largest group of enzymes in the whole genome and are involved in synthesizing various secondary metabolites, including plant defense substances, stress resistance substances, fatty acids, plant hormones that are essential for the plant life cycle [5-7]. Also, plant CYP plays a pivotal role in the production of various natural products in plants and is an essential component in metabolic engineering for biosynthesis of bioactive natural products [8, 9]. CYP71AV1 has been applied to synthetic engineering of artemisinin, a precursor for antimalarial drugs, and three CYP716A enzymes (*i.e.*, CYP716A12, CYP716A47, and CYP716A53v2) have been used to synthetic engineering of triterpenoid saponin [8, 10].

**Fig. 1:**
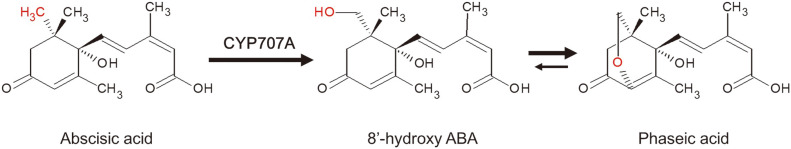
ABA catabolic pathway. Red character indicates ABA 8’- hydroxylation site.

The CYP707A used in this study is known as ABA 8'-hydroxylase in plants, is involved in the response of environmental stresses, seed germination, root elongation and fruit ripening, through the abscisic acid (ABA) catabolism [11-14]. In this catabolic process, CYP707A catabolizes ABA into 8'-hydroxy ABA, and the resulting 8'-hydroxy ABA is spontaneously converted to phaseic acid (PA) (Fig. 1) [15]. Although PA is a catabolite of ABA, PA acts as an important signal molecule in plant physiology and environmental adaptation along with ABA in higher plants [16]. Moreover, PA acts as a neuroprotective molecule that can reversibly inhibit glutamate receptors during ischemic brain injury in the mice [17]. According to these reports, the PA is expected to be a valuable substance not only for the agricultural but also for the pharmaceutical industry.

Most plant CYPs are membrane-bound proteins that are primarily expressed in the endoplasmic reticulum, which differs from prokaryotic soluble cytosolic CYPs [18]. The lipophilic sequences of the membrane-bound proteins caused aggregation and hindered protein crystallization and structure determination. To improve the solubility of CYP proteins, studies have been conducted to remove or modify residues in the N-terminal transmembrane anchor region based on the hypothesis that the hydrophobicity of the N-terminal anchor prevents efficient expression in bacteria. Although some studies observed that truncation of the N-terminal region allowed successful expression, increased expression through N-terminal truncation is still difficult to predict because this proline-rich region plays an essential role in correct folding [19].

In this study, we tried to establish a system that efficiently expresses plant CYP707A at cytosolic fraction in *E. coli* and used it to produce the PA. Four CYP707A genes were isolated from hot pepper and heterologously expressed in *E. coli*. Without N-terminus modification, we found only CaCYP707A2 could be functionally expressed as a soluble form in cytosolic fraction. To increase the efficiency of the catalytic activity of CaCYP707A2, a preference investigation with redox partners showed much higher interaction with plant CaCPR than with other redox partners. Finally this study confirmed the generation of products according to the reaction time in the CaCYP707A2 from the soluble fraction with high industrial applicability. This is the first report of expressed plant CYP as a soluble form in the cytosolic fraction of *E. coli*.

## Materials and Methods

### Materials

(+)-ABA, glucose-6-phosphate, glucose-6-phosphate dehydrogenase, NADP^+^, isopropyl β-D1-thiogalactopyranoside (IPTG), spinach ferredoxin reductase (FdR), and spinach ferredoxin (Fdx) were obtained from Sigma-Aldrich (USA). Recombinant rat NADPH-cytochrome P450 reductase (CPR) and CaCPR was expressed in *E. coli* and purified as previously described [20, 21]. All other chemicals were analytical grade.

### Amino Acid Sequence and Structure Analysis

In a previous study, we isolated four *CaCYP707A* genes (*CaCYP707A1*, *CaCYP707A2*, *CaCYP707A3*, and *CaCYP707A4*) from the cDNA of hot pepper [22]. For further investigation of the structurally important motifs and 3D structure prediction, the amino acid sequences deduced from the four *CaCYP707A* genes were analyzed. Accession numbers of genes used for analysis are listed in Table 1. Multiple amino acid sequence alignments were performed using Sequence Manipulation Suite: Color Align Conservatioin (https://www.bioinformatics.org/sms2/color_align_cons.html) and Clustal W [23]. Prediction of transmembrane was performed using the TMHMM Server ver. 2.0 (http://www.cbs.dtu.dk/services/TMHMM/). The protein structural prediction was obtained from the AlphaFold Protein Structure Database [24].

### Heterologous Expression in *E. coli* and Purification of CaCYP707A

**Fig. 2:**
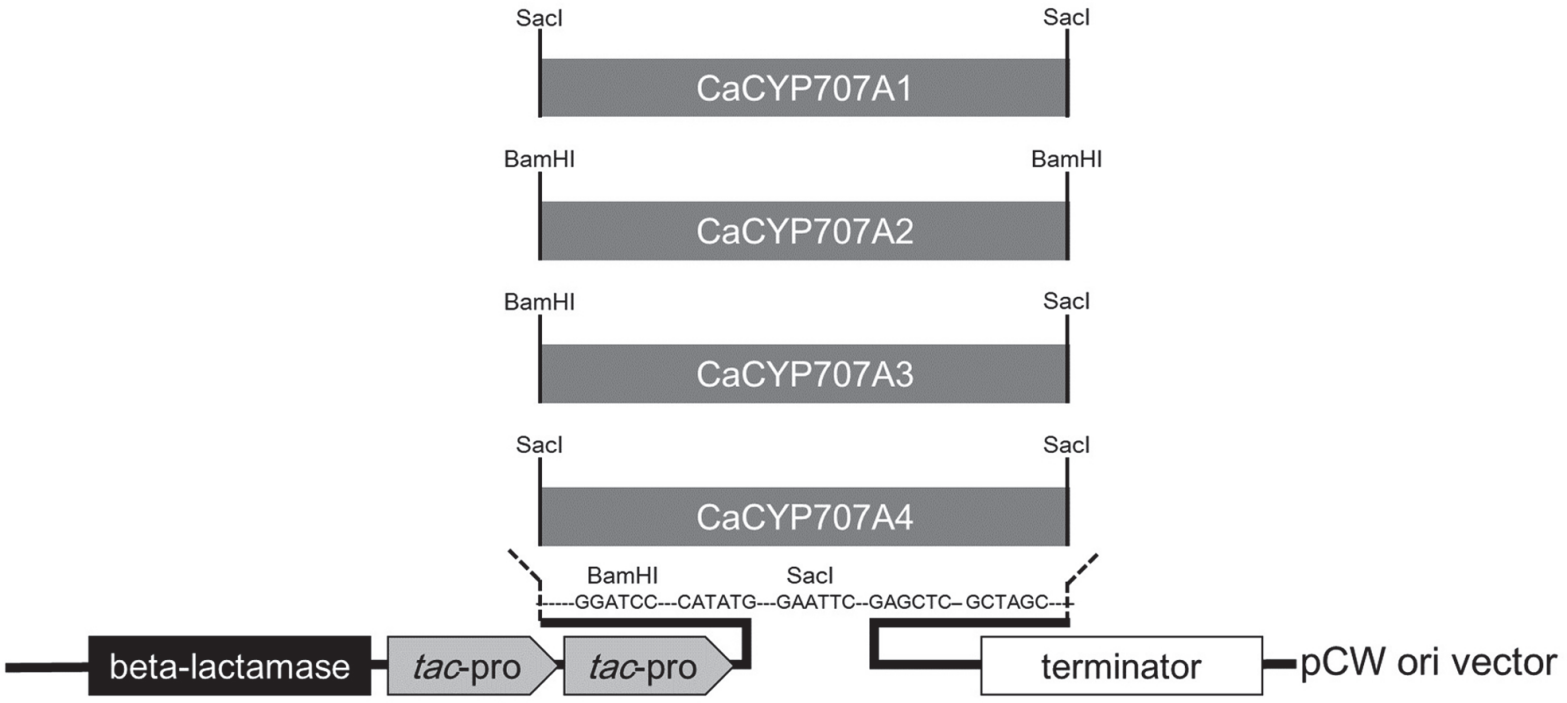
Construction of pCW expression vector for CaCYP707A. *tac*-pro: *tac* promoter, beta-lactamase: ampicillin resistance gene.

In a previous study, four *CaCYP707A* genes were transformed into *E. coli*, and the ABA 8'-hydroxylation activity was confirmed [22]. These transformed *E. coli* cells were used to investigate the soluble/membrane expression of heterologously expressed CaCYP707A proteins and its redox partner preference. The *E. coli* strains and plasmid information used for heterologous expression are listed in Table 1. Vector constructs for protein expression are shown in Fig. 2. The transformed *E. coli* cells were incubated in a Luria-Bertani (LB) medium containing 50 μg/ ml of ampicillin. To express the protein, LB liquid cultures were transferred into 1L of Terrific broth (TB) (Thermo Fisher Scientific, USA) that containined additives [i.e, 50 μg/ml of ampicillin, 1 mM of thiamine, 0.5 mM of δ-aminolevulinic acid, and trace elements (100 mM FeCl_3_∙6H_2_O, 10 mM ZnCl_2_∙4H_2_O, 10 mM CoCl_2_∙6H_2_O, 10 mM Na_2_MoO_4_, 6.8 mM CaCl_2_∙2H_2_O, 7.4 mM CuCl_2_, 8 mM H_3_BO_3_ and 1% HCl)] and incubated at 37°C and 180 ×*g* until reaching the optimal density at a wavelength of 600 nm. Expression was induced with 1 mM of IPTG. The expression cultures were incubated at 28°C and 200 ×*g* for 32 h.

To confirm the protein expression in cultured *E. coli*, 1 ml of the culture was collected and centrifuged at 13,000 ×*g* for 1 min. The cell pellet was resuspended in TES buffer (100 mM Tris-HCl, pH 7.6, 500 mM sucrose and 0.5 mM EDTA) and lysed by brief sonication (1 sec on, 10 sec off) repeated five times. Soluble part proteins were obtained from the supernatant by centrifuging the suspension at 13,000 ×*g* for 5 min, and insoluble part proteins were obtained by second resuspending in TES buffer. The presence of the protein of interest in both parts was confirmed using 8% sodium dodecyl sulfate polyacrylamide gel electrophoresis (SDS-PAGE) gel.

To isolate the proteins, the *E. coli* cells containing each expression plasmid were harvested by centrifugation (5,000 g, 15 min, 4°C). The cell pellet was resuspended in TES buffer and sonicated (5 sec on, 30 sec off) 10 times to lyse. To remove cell debris, the sonicated fraction was centrifuged at 5,000 g (15 min, 4°C) and the resulting supernatant was ultracentrifuged at 100,000 g (120 min, 4°C). The soluble cytosolic fraction was collected and stored at -80°C until use. The membrane fraction was resuspended in 100 mM potassium phosphate buffer (pH 7.4) containing 20% glycerol (v/v) and subsequently homogenized with a dounce homogenizer. The homogenized membrane fraction was stored at -80°C until use. The presence of the protein of interest in both separated fractions was analyzed on 8% SDS-PAGE gel.

### Spectroscopic Analysis

The CaCYP707A1, CaCYP707A2, CaCYP707A3, and CaCYP707A4 concentrations in both the membrane fraction and the soluble fraction were calculated using the CO difference spectra as previously described [25]. Sodium dithionite was added to reduce the CYPs, and CO gas was injected to create a CO saturated condition. UV-visible spectra were recorded for absorbance from 400 to 500 nm using a Shimadzu UV-1601 spectrophotometer.

### Substrate Binding Titrations

Spectral determination of the *K_D_* values for the binding of (+)-ABA substrates to the CaCYP707A2 was performed as previously described [26]. CaCYP707A2 was diluted to 0.5 μM in 100 mM potassium phosphate buffer (pH 7.4) and divided into two 1.0 ml glass cuvettes and a baseline was set. The (+)-ABA were dissolved in dimethylsulfoxide, and the final dimethylsulfoxide concentrations were < 1% (V/V). For spectrophotometric titration, ABA was added to CaCYP707A2 at a concentration ranging from 0 to 80 μM, a difference in absorbance between 350 to 500 nm was recorded using a Shimadzu UV-1601 spectrophotometer. The absorption difference between 389 and 420 nm was plotted against the substrate concentration. The binding affinities were estimated using the Graph-Pad Prism software as previously described (Graph-Pad, USA) [27].

### Analysis of Enzymatic Activities of CaCYP707A2

Reaction mixtures were constructed to confirm the ABA hydroxylation activity of CaCYP707A protein. The reaction mixtures had a total volume of 250 μl and contained 50 pmol CaCYP707A2 (both the membrane fraction and the soluble fraction), 100 μM ABA, 100mM potassium phosphate buffer (pH 7.4), and an NADPH-generating system (final concentration of 10 mM glucose-6-phosphate, 0.5 mM NADP^+^, and 1.0 IU of yeast glucose-6-phosphate dehydrogenase/ml). Three redox partners were used to compare the CaCYP707A2 catalyzed reactions: rat CPR (100 pmol), CaCPR (100 pmol), FdR/Fdx (0.1 units of FdR/ 20 μg of Fdx). For the membrane fraction protein, the reactions were performed at 28°C for 4 h. For soluble fraction proteins, the reactions were performed at 28°C for 15, 30, 45, 60 and 120 min. After incubation, the reactions were stopped by adding 25 μl of 1 N HCl. The reaction mixtures were extracted with 500 μl of ice-cold ethyl acetate. The products were extracted with centrifugation at 3,000 rpm for 5 min. The upper layer was transferred to a clean test tube, this process was repeated two more times. The solvent was evaporated under a gentle stream of nitrogen. The dried products were redissolved in 180 μl of mobile phase solution and transferred to a glass vial for HPLC analysis.

These dissolved products were injected into a Gemini C18 column (4.6 mm × 150 mm, 5 μm, Phenomenex) equipped with a HPLC system (Shimadzu Nexera LC-40D XS, Japan) and UV detector. The mobile phase solution consisted of water that contained 0.05% acetic acid and 37% methanol (v/v). The flow rate was 1.0 ml/min and the absorbance was measured at 254 nm.

### Statistical Analysis

All experiments were performed three times. The data were presented as means with ± standard deviation of three determinations.

## Results

### Analysis of Hot Pepper CYP707A Amino Acid Sequence

**Fig. 3:**
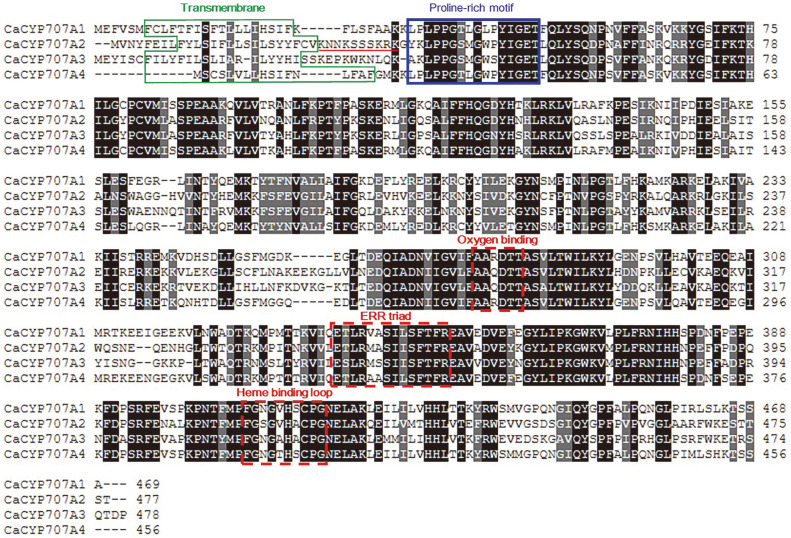
Alignment of deduced amino acid sequence of *CaCYP707A* genes from *C. annuum*. Identical amino acid residues are shaded in black and similar amino acid residues are shaded in gray. Predict transmembrane domains are indicated by green box. Proline-rich motif are indicated by blue box. The putative oxygen binding motif and ERR triad, and heme binding are indicated by red boxes.

ABA 8'-hydroxylase is an enzyme that catabolize ABA and plays a key role in PA production [28]. In a previous study, we isolated four ABA 8'-hydroxylase genes from hot pepper (*CaCYP707A1*, *CaCYP707A2*, *CaCYP707A3*, and *CaCYP707A4*) [22]. By comparing the amino acid sequences deduced based on the DNA sequence information of the isolated genes, three highly conserved regions (oxygen binding site, E-R-R triad site, and heme-binding loop) essential for CYP catalytic activity were identified (Fig. 3). The transmembrane domain located at the N-terminus was predicted through the TMHMM Server, and their sequence similarity was revealed to be very low.

### Heterogeneous Expression of CaCYP707A in *E. coli*

**Fig. 4:**
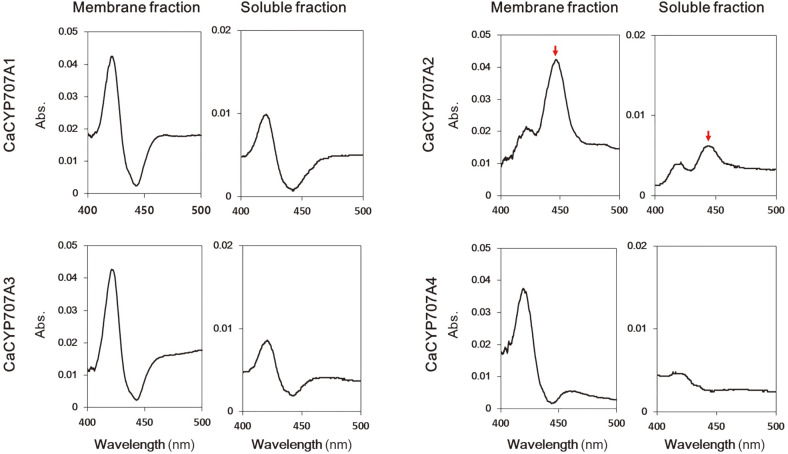
CO-binding difference spectra of CaCYP707A proteins from membrane fraction and soluble fraction. CYP-specific 450 nm peaks were indicated by red arrow.

CaCYP707A proteins from hot pepper were targeted to express CYP proteins with stable activity in a heterologous expression system using *E. coli*. Various factors such as temperature, incubation time, aeration, and medium supplementation affect the expression of CYP proteins, and low-temperature incubation has been reported to produce stable protein folding without aggregation [29]. To estimate the expressed a CYP protein, the CO difference spectrum was measured for each incubation temperature and incubation time in *E. coli*, which is transformed with the vector expressing the CaCYP707A proteins. The reduced-CO difference spectrum of CYP proteins is a feature of CYP proteins that is widely used for qualitative and quantitative estimation [30]. The protein expression at different induction times (20, 24, 30, 36, 42, and 48 h) in *E. coli* cells were performed at several temperatures (25, 28, 32, and 37°C). The optimal expression levels of CaCYP707As were observed at 28°C with about 32 h culture (data not shown). Base on this, the expression of CaCYP707A proteins were investigated in the membrane fraction and soluble fraction isolated from *E. coli* (Fig. 4). In case of CO difference spectrum of the CaCYP707A1 membrane fraction, the Soret absorption peak was observed at 420 nm but not at 450 nm. Similar results were confirmed in the membrane fractions of CaCYP707A3 and CaCYP707A4, indicating that the isolated CaCYP707A1, CaCYP707A3 and CaCYP707A4 proteins can easily be deactivated. Meanwhile, the membrane fraction of CaCYP707A2 showed a clear absorption peak at 450 nm. The estimated expression of the enzyme using the CO difference spectrum was 29.4 nM for CaCYP707A2, but 0.3 nM and 2.4 nM for CaCYP707A1 and CaCYP707A4, respectively. CaCYP707A3 was not identified. In addition, CaCYP707A2 showed an absorption peak of 450 nm in the soluble fraction. However, CO difference of spectrum of other three CaCYP707As did not show. Expression in the soluble fraction was the highest at 3.2 nM for CaCYP707A2, but at 0.3 nM for CaCYP707A3. CaCYP707A1 and CaCYP707A4 from the soluble fraction were not identified. Therefore, CaCYP707A2 was selected for further experiments.

**Fig. 5:**
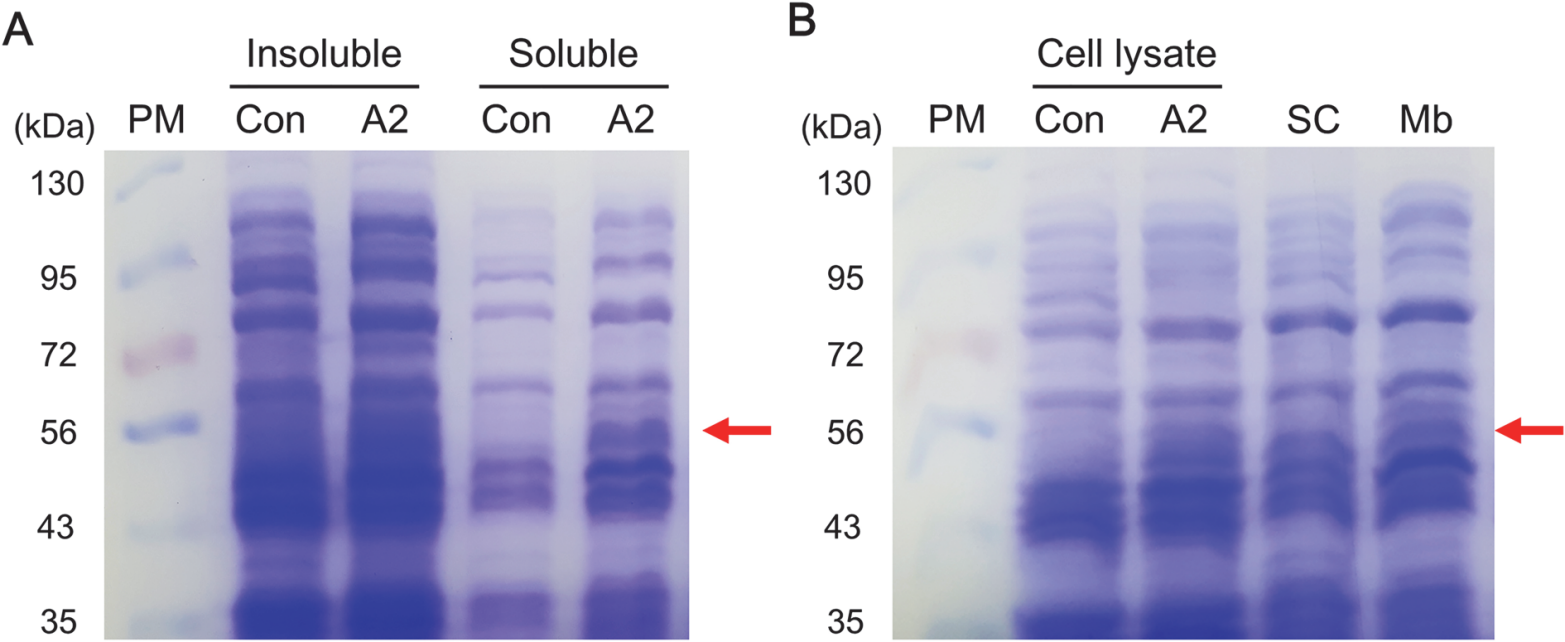
SDS-PAGE analysis of the CYP707A2 proteins. The presence of the CYP707A2 protein (54 kDa) was confirmed by staining an 8% SDS-PAGE gel with Coomassie Blue. (A) SDS-PAGE of insoluble and soluble proteins of *E. coli* cultures. (B) SDS-PAGE of total cell lysates and fractionated lysates from *E. coli* cell expressing CaCYP707A2. PM: protein morecular weight marker, Con: empty vector harboring *E. coli*. A2: CaCYP707A2 expressed *E. coli*. SC: soluble cytosolic fraction from CaCYP707A2 expressed *E. coli*. Mb: membrane fraction from CaCYP707A2 expressed *E. coli*. The red arrow indicates the protein of interest.

From the CO difference spectrum results, the expression of CaCYP707A2 was estimated in the cytosolic and membrane fractions of *E. coli*. To confirm this, the cultured *E. coli* was prepared under non-denaturing conditions and the presence of protein was evaluated using SDS-PAGE (Fig. 5). The CaCYP707A2 protein was found in both soluble and insoluble parts (Fig. 5A). Additionally, the presence of CaCYP707A2 protein was found in the same position as *E. coli* cell lysate in the separated soluble cytosolic fraction and membrane fraction (Fig. 5B). This indicates that it is consistent with the estimated expression result using the CO difference spectrum.

**Fig. 6:**
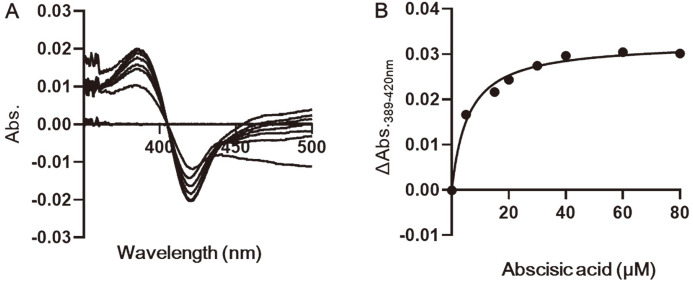
Binding titration of CaCYP707A2 with ABA. (A) The 0.5μM CaCYP707A2 was divided into each of two glass cuvettes and baseline was set. (+)-ABA was added to the cuvette at ranging from 0 to 80 μM, and difference spectra was recorded between 500 nm and 350 nm. With increasing concentrations of (+)-ABA, spectra were increased at 386 nm and decreased at 419 nm. (B) The difference between absorbance at 389 nm and 420 mm were recorded using the dual wavelength mode, as a function of (+)-ABA.

To confirm whether (+)-ABA can bind to CaCYP707A2, a spectral binding titration assay was performed (Fig. 6). By measuring the spectrum for the gradual increase in ABA concentration using CaCYP707A2, a type I difference spectrum with a peak at 386 nm and a trough at 419 nm was observed. The spectrally determined dissociation constants (*K_D_*) of CaCYP707A2 to ABA was 5.8 ± 0.93 μM.

### Dependence of CaCYP707A2 Activity on the Redox Partner

**Fig. 7:**
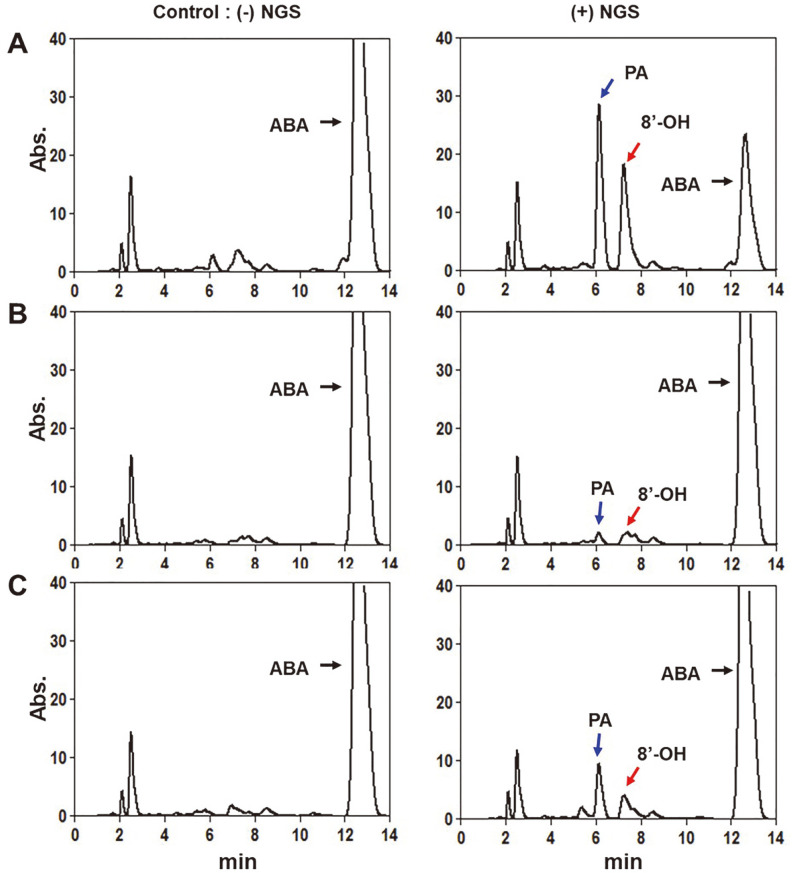
HPLC analysis of ABA metabolites produced by CaCYP707A2. CaCYP707A2 protein from membrane fraction was incubated with (+)-ABA and CaCPR (A), rat CPR (B), and FdR/Fdx (C) in the absence (left; –NGS) or presence (right; +NGS) of a NADPH-generating system. Metabolites produced by CaCYP707A2 catalytic activity were analyzed by HPLC. The peaks marked by red and blue arrows are 8’-hydroxy ABA (8’-OH) and phaseic acid (PA), respectively, whereas the peaks marked by black arrows are abscisic acid (ABA).

The CYP catalysis requires electron-transferring redox proteins, which is one of the factors determining the rate of CYP catalysis [31]. Therefore, studies have attempted to identify the preferred redox partner according to CYP as a strategy for optimizing catalysis [32, 33]. The catalytic activity of CaCYP707A2 was investigated using redox partners including CaCPR, rat CPR, and FdR/Fdx, to determine the best redox partner to achieve the optimal electron transfer pathway in catalysis (Fig. 7). When CaCYP707A2 was incubated with CaCPR, 8'-hydroxy ABA and PA were produced due to the ABA hydroxylation activity. Although 8'-hydroxy ABA and PA were detected in the catalytic reaction using rat CPR and FdR/Fdx, the amounts were much smaller than those observed after using CaCPR. These results indicate that interaction with redox partners affects the catalysis of CaCYP707A2, and CaCPR is the most preferred partner.

### ABA Hydroxylation Activity of CaCYP707A2 Protein

**Fig. 8:**
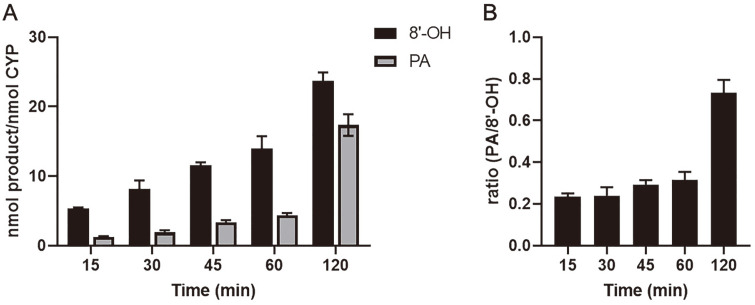
Production of ABA metabolites by CaCYP707A2 from soluble fraction over time. CaCYP707A2 protein from soluble fraction was incubated with (+)-ABA and CaCPR for indicated time (15-120 min). Production of ABA metabolite over time was calculated from area on chromatogram of HPLC results (A). The PA generation ratio over time was calculated from the product amount of 8'-hydroxy ABA plus PA (B). The values are represented as the mean with SD of triple measurements. 8’-OH: 8’-hydroxy ABA, PA: phaseic acid.

In the CO difference spectrum results, the CYP-specific absorption peak of 450 nm was observed not only in the membrane fraction, but also in the soluble fraction of *E. coli* where CaCYP707A2 was heterologously expressed. Additional experiments were performed with a time course based on reaction time to investigate whether CaCYP707A2 protein from the soluble fraction has ABA hydroxylation activity and whether incubation time has an effect on the production of 8'-hydroxy ABA and PA (Fig. 8). The redox partner for electron transfer in the catalytic reaction was used CaCPR, which showed the highest interaction with CaCYP707A2. In the case of 8'-hydroxy ABA, the amount of 8'-hydroxy ABA gradually increased as the reaction time increased. PA also showed a gradually increase as the reaction time increased until 2 h. The PA generation ratio of the reaction products was 0.2 to 0.3 when incubated for 15 to 60 min. Interestingly, after 2 h of incubation, it showed an increase over 0.7. These results indicate that the CaCYP707A2 protein from the soluble fraction has the same ABA hydroxylation activity as the CaCYP707A2 protein from the membrane fraction, and that the conversion ratio of 8'-hydroxy ABA to PA increases with time. Taken together, these findings suggest that the ABA hydroxylation activity of CaCYP707A2 is applicable to catalytic reaction systems for PA production.

## Discussion

Plant CYPs are involved in the biosynthetic pathway of various anticancer and therapeutic agents derived from plants, such as Taxol, terpenoid indole alkaloid family substances, and a precursor of artemisinin (a powerful antimalarial drug) [34, 35]. The application of plant CYPs to produce these high-value metabolites is an important goal in metabolic engineering.

However, two characteristics of plant CYPs often limit widespread application in metabolic engineering. First, CYP catalysis requires additional redox partners for sustained electron transport [36]. When constructing a metabolic engineering system through heterologous expression, the lack of information on redox partners and low accessibility to CYP hinder the catalytic reaction [4]. In the field of metabolic engineering, studying the interaction between CYP and redox partners to increase the efficiency of catalysis is important. Bacterial FdR/Fdx was used to find the optimal redox partner that interacts in CYP catalysis, and the results showed that depending on the CYP, using of a non-homologous surrogate redox partner can be helpful for optimizing the catalysis reaction [32, 37]. To increase the interaction efficiency of CYP and its redox partner, rat CPR was fused with CYP and the expression site of rat CPR in bacteria was adjusted for efficient substrate delivery [38, 39]. In addition, the characteristics of plant CPR were also investigated to understand the activity and role of CPR in plants [21]. In this study, the ABA hydroxylation activity of CaCYP707A2 produced more products with CaCPR than with rat CPR and FdR/Fdx (Fig. 7). These results indicate that selection of optimal redox partners is important to increase the efficiency of CYP catalysis, suggesting that the application of CaCPR to metabolic engineering using plant CYP is more effective.

Second, plant CYPs tend to exhibit low stability, low activity, and low expression levels in non-native hosts. Statistical data showed that out of over 250 plant CYPs, about 40% were difficult to express in yeast [8]. In this study, four CaCYP707A proteins were heterologously expressed in *E. coli*. In the CO difference spectra results of the CaCYP707A1, CaCYP707A3, and CaCYP707A4 proteins, the CYP-specific absorption peak of 450 nm was poorly observed (Fig. 4). These results suggest that heterologous expressions of CaCYP707A1, CaCYP707A3, and CaCYP707A4 proteins in *E. coli* have low stability and low activity. In contrast, CaCYP707A2 showed a CYP-specific absorption peak 0f 450 nm (Fig. 4). Notably, this result was observed not only in the membrane fraction but also in the soluble fraction. In addition, the CaCYP707A2 protein that was isolated from this soluble fraction was confirmed to have ABA hydroxylation activity related to 8'-hydroxy ABA and PA production (Fig. 8). This suggests that there is a difference between CaCYP707A2 and other CaCYP707A proteins that plays a crucial role in the stability of expression and maintenance of enzyme activity.

In the amino acid alignment results among CaCYP707A1, CaCYP707A2, CaCYP707A3, and CaCYP707A4, many regions were highly similar, but the N-terminal region was not similar to each other (Fig. 3). In particular, it is necessary to note to the amino acid sequence between the predicted transmembrane region of each protein (green box) and the proline-rich motif (blue box) (Fig. 3). The sequences for this part of CaCYP707A1, CaCYP707A3, and CaCYP707A4 contain hydrophobic amino acid residues, whereas the sequence of CaCYP707A2 consists mostly of hydrophilic amino acid residues (underlined in red) (Fig. 3). Studies conducted to obtain a catalytically active protein expressed in the cytoplasm of *E. coli* showed that introducing positive charges into the N-terminal linker region can lower the proportion of the protein localized in the inner membrane of *E. coli* [40].

**Fig. 9:**
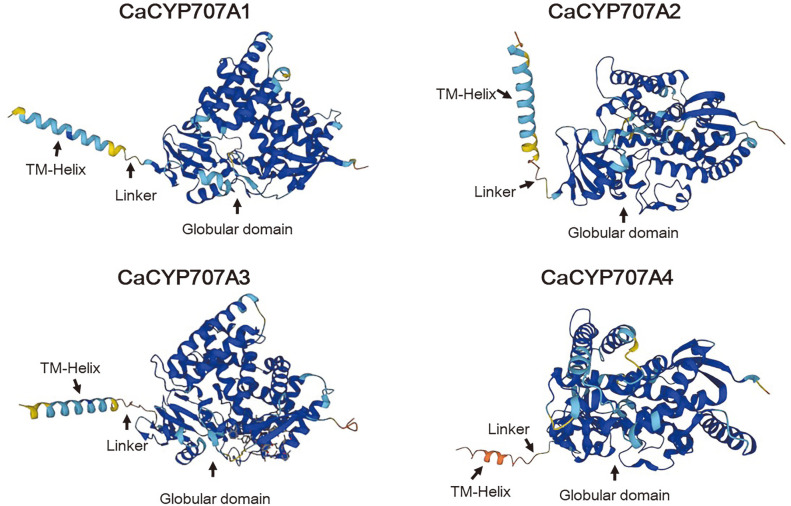
AlphaFold predicted structures of full-length CaCYP707A1, CaCYP707A2, CaCYP707A3, and CaCYP707A4. TM-Helix: transmembrane domain helix.

The hydrophobic sequence length of the transmembrane domain and the distribution of amino acid residues may adopt different orientations or tilt angles when binding to the membrane [41]. The angle of the transmembrane domain affects the membrane binding of CYP proteins [42]. To further understand the protein structure, AlphaFold was used to predict the 3D structures of the CaCYP707A proteins (Fig. 9) [24]. In general, eukaryotic membrane-bound CYPs have a transmembrane-helix domain that is located at the N-terminus, a linker region, and a globular domain structure that performs a catalytic action [42]. The structures of the predicted CaCYP707A proteins were also observed similarly to the reported structure of the transmembrane-helix domain, linker region, and globular domain. In the 3D structure predicted through the AlphaFold, however, the transmembrane domain of the CaCYP707A2 protein shows a different tilt angle than other CaCYP707A proteins (Fig. 9). This result suggests that the distribution of hydrophilic and hydrophobic amino acid residues in the linker region of CaCYP707A2 protein may affect the membrane binding interaction and solubility of the CYP protein by changing the tilt angle of the transmembrane domain. Further experiments are needed to determine whether the linker region affects CYP solubilization and expression stability enhancement. The replacement of the linker region of membrane anchor proteins and additional sequence modifications will increase the availability for stable activity and expression of a variety of membrane-bound CYPs, including plant CYPs.

## Figures and Tables

**Table 1:** *E. coli* strains and plasmids used in this work.

CYPs	Accession number	*E. coli* strain	Plasmid
CaCYP707A1	MT198680	Rosetta (DE3) pLysS	pCW ori(+)
CaCYP707A2	JQ828939	Rosetta (DE3) pLysS	pCW ori(+)
CaCYP707A3	MT198681	Rosetta (DE3) pLysS	pCW ori(+)
CaCYP707A4	MT198682	Rosetta (DE3) pLysS	pCW ori(+)
